# “Micropersonality” traits and their implications for behavioral and movement ecology research

**DOI:** 10.1002/ece3.7275

**Published:** 2021-02-22

**Authors:** Joseph D. Bailey, Andrew J. King, Edward A. Codling, Ashley M. Short, Gemma I. Johns, Ines Fürtbauer

**Affiliations:** ^1^ Department of Mathematical Sciences University of Essex Colchester UK; ^2^ Department of Biosciences College of Science Swansea University Swansea UK

**Keywords:** animal personality, *Gasterosteus aculeatus*, interindividual differences, stickleback fish, tracking, trajectories

## Abstract

1. Many animal personality traits have implicit movement‐based definitions and can directly or indirectly influence ecological and evolutionary processes. It has therefore been proposed that animal movement studies could benefit from acknowledging and studying consistent interindividual differences (personality), and, conversely, animal personality studies could adopt a more quantitative representation of movement patterns.
2. Using high‐resolution tracking data of three‐spined stickleback fish (*Gasterosteus aculeatus*)*,* we examined the repeatability of four movement parameters commonly used in the analysis of discrete time series movement data (time stationary, step length, turning angle, burst frequency) and four behavioral parameters commonly used in animal personality studies (distance travelled, space use, time in free water, and time near objects).
3. Fish showed repeatable interindividual differences in both movement and behavioral parameters when observed in a simple environment with two, three, or five shelters present. Moreover, individuals that spent less time stationary, took more direct paths, and less commonly burst travelled (movement parameters), were found to travel farther, explored more of the tank, and spent more time in open water (behavioral parameters).
4. Our case study indicates that the two approaches—quantifying movement and behavioral parameters—are broadly equivalent, and we suggest that movement parameters can be viewed as “micropersonality” traits that give rise to broad‐scale consistent interindividual differences in behavior. This finding has implications for both personality and movement ecology research areas. For example, the study of movement parameters may provide a robust way to analyze individual personalities in species that are difficult or impossible to study using standardized behavioral assays.

## Graphical abstract

We use high‐resolution tracking data of three‐spined stickleback fish (*Gasterosteus aculeatus*) to examine the repeatability of movement parameters commonly used in the analysis of discrete time series movement data and behavioral parameters commonly used in animal personality studies. We show that movement and behavioral parameters are broadly equivalent, capturing similar interindividual variation. We suggest that movement parameters can be viewed as “micropersonality” traits that give rise to broad‐scale consistent interindividual differences in behavior.

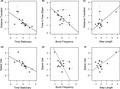

## 1 INTRODUCTION

Understanding and predicting animal space use is central to the advancement of ecological research (Kanagaraj et al., 2013; Nathan et al., 2008). Mechanistic models of animal movement tend to assume animal movement is either fixed or else flexible with respect to environmental heterogeneity or uncertainty (Fofana & Hurford, 2017; Grunbaum, 1998; Moorcroft, 2012). Studies supporting flexible movement strategies are growing; animals have the ability to make choices about their environment and respond to stimuli using their various sensory mechanisms (e.g., Ben‐Ari & Inbar, 2014; Hopkins, 2016; Lemasson et al., 2009), and movement paths can emerge from interactions with heterogeneous landscapes (e.g., Lima & Zollner, 1996; Sueur et al., 2011). However, to predict population dynamics and the emergence of ecological patterns from individual behavior requires thorough consideration of inter‐ and intraspecific variation in movement patterns and not only the spatial structure of the landscape (Belgrad & Griffen, 2018; Getz et al., 2018; Morales & Ellner, 2002; Sih et al., 2018; Spiegel et al., 2017).

Variation in movement patterns can be linked to interindividual differences in, for example, exploratory tendency (e.g., Herborn et al., 2010; King et al., 2013) where some individuals explore fast and superficially, while others explore slowly and more thoroughly (e.g., Dingemanse et al., 2002; Guillette et al., 2009), or boldness, where bolder individuals are more likely to move toward (or less likely to retreat from) threat or risk (e.g., Fürtbauer et al., 2015; Williams et al., 2012). Indeed, such “personality traits” have implicit movement‐based definitions and can directly or indirectly influence ecological and evolutionary processes (Spiegel et al., 2017; Wolf & Weissing, 2012). It has therefore been proposed that movement studies could benefit from acknowledging and studying consistent interindividual differences, and, conversely, animal personality studies could adopt a more quantitative representation of movement patterns (Spiegel et al., 2017; Webber & Vander Wal, 2018).

Despite the calls for synergy between personality and movement ecology research (e.g., Getz et al., 2018; Spiegel et al., 2017), studies using individual‐level data to build data‐ or theory‐driven movement models are rare. However, with advances in tracking technologies allowing researchers to record many individuals' movement simultaneously in the wild (Kays et al., 2015; King et al., 2018) and software enabling the identification and tracking of individuals in the laboratory (Krause et al., 2013; Romero‐Ferrero et al., 2019), such work has become possible. For example, during the process of undertaking and writing this study, several works examining individual variation in animal movements using telemetry data have been published (Harris et al., 2020; Harrison et al., 2019; Hertel et al., 2020) and this behavioral type‐based approach to study movement ecology allows for better understanding of community and population responses (e.g., Harris et al., 2020; Morales & Ellner, 2002).

**FIGURE 1:**
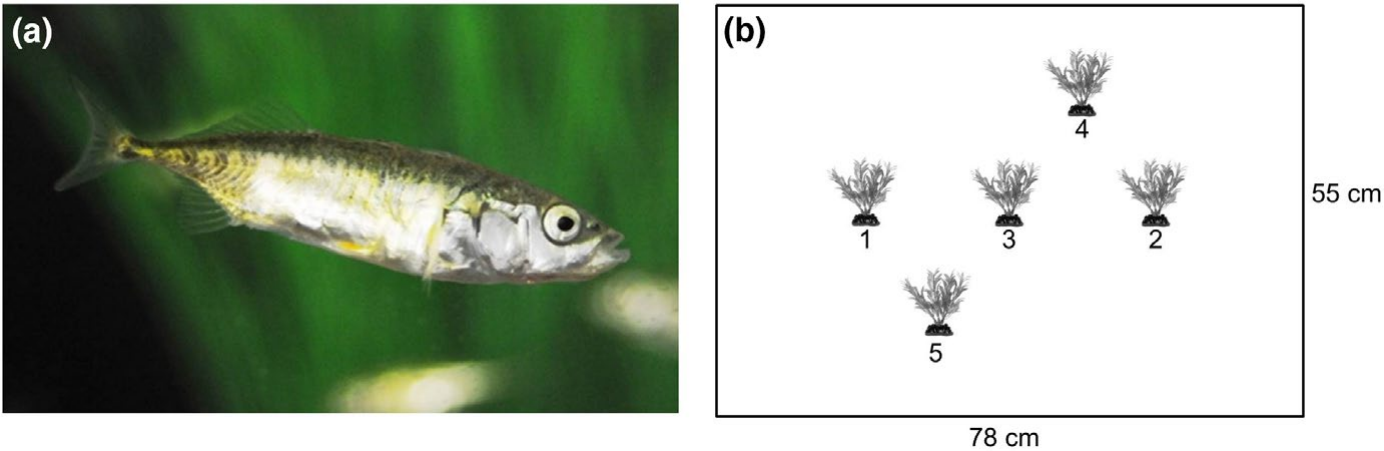
(a) Female *Gasterosteus aculeatus*; (b) Set‐up. Fish were observed in a 78 × 55 × 16 cm plastic tank, filled to 12 cm with water. The fish were observed with either two shelters (plants 1 and 2), three shelters (plants 1–3), or five shelters (plants 1–5)

Here, we study three‐spined stickleback fish (*Gasterosteus aculeatus*, Figure 1a), a classic behavioral model species (Bell, 1995) that exhibits interindividual differences in behavior (e.g., Bell, 2005; Dingemanse et al., 2007; King et al., 2013) and flexibility with respect to environmental changes (e.g., Bell & Sih, 2007; Fürtbauer et al., 2015; Hansen et al., 2016). Using high‐resolution tracking data, we examined the repeatability of four movement parameters commonly used in the analysis of discrete time series movement data (time stationary, step length, turning angle, burst frequency) and four behavioral parameters commonly used in animal personality studies (distance travelled, space use, time in free water, and time near objects). We chose these movement parameters because they constitute the basis of movement path analyses in movement ecology research (e.g., Ben‐Ari & Inbar, 2014; Hopkins, 2016; Kane et al., 2004; Kareiva & Shigesada, 1983; Lemasson et al., 2009; Lima & Zollner, 1996; Sueur et al., 2011). We chose these behavioral parameters as they are commonly studied in this species to investigate activity and exploratory behavior (e.g., Dzieweczynski & Crovo, 2011; Jolles et al., 2018; King et al., 2013; Mamuneas et al., 2015), and such descriptors are repeatable and related to physiological measures in our study population (Fürtbauer et al., 2015).

We expected fish to show consistent interindividual differences in movement and behavioral parameters, across time and context (i.e., in different environments, sampled repeatedly). Furthermore, because consistent interindividual differences in activity and exploration (i.e., personality traits) have implicit movement‐based definitions (Spiegel et al., 2017), we hypothesized the two approaches—quantifying movement and behavioral parameters—would be broadly equivalent, whereby interindividual differences in precise movement characteristics give rise to broad‐scale interindividual differences in behaviors. If true, we propose that interindividual differences in movement parameters could be usefully viewed as “micropersonality” traits.

## 2 METHODS

### 2.1 Subjects and housing

Wild three‐spined sticklebacks (*Gasterosteus aculeatus*) (Figure 1a) were caught from Swansea University campus pond, UK. Fish were kept in a holding tank (300 × 390 × 1,220 mm) containing gravel substrate, plants, and driftwood for 2 weeks prior to behavioral testing at a consistent temperature of 16°C and with 8 hr:16 hr light:dark photoperiod regime. Fish were fed bloodworms (*Chironomus* sp.) daily. During behavioral testing, fish were kept in individual 2.8 L gravel‐lined, aerated tanks, with visual access to neighbors.

### 2.2 Fish observations

Fish were filmed using a Panasonic HDC‐SD60 HD video camera (Panasonic Corporation of North America) mounted on a custom‐built metal frame (1 × 1 × 1.5 m) surrounded by white sheeting (PhotoSEL BK13CW White Screen). Four photographer's lights (each with 4 × 25w 240v 6400K True Day light bulbs) lit the arena from outside the white sheet, dispersing light evenly. Fish were observed for 15 min after being placed in the bottom left‐hand corner of an opaque plastic tank, 78 × 55 × 16 cm, which was lined with white gravel and filled with water to 12 cm (and water was changed after each trial). Fish were observed with either two, three, or five plastic plants at fixed positions (Figure 1b) representing increasingly heterogeneous environment and were repeat tested 1 week later in the reverse order (*n* = 15 fish, *n* = 6 trials per fish, total *N* = 90). Data for *n* = 1 fish in week 1 could not be fully tracked from video, resulting in an overall sample of *N* = 87.

### 2.3 Fish trajectory data

Video recordings were processed using IDTracker (Perez‐Escudero et al., 2014) to generate *x*, *y* coordinates for fish, frame by frame (25 Hz recording). Movement was therefore considered to be formed by a discrete step‐turn process. Data were then manually checked, and a value of 5 mm/s was chosen as a threshold to determine movement, which represented movement across frames of less than a pixel (Duteil et al., 2016). A subsampling rate of 2.5 Hz was used to prevent false large turns which can occur due to the processing of the video recording (Delcourt et al., 2013). The movement threshold and subsampling rates are in essence arbitrary values but were chosen to retain as much information about the movement path, while minimizing any causal effects such smoothing can have on characteristics of movement trajectories (Bailey et al., 2020; Benhamou, 2014; Bovet & Benhamou, 1988; Codling & Hill, 2005; Gurarie & Ovaskainen, 2011); different combinations of thresholds and subsampling did not affect our findings (Figures S2–S10).

### 2.4 Movement and behavioral parameters

**FIGURE 2:**
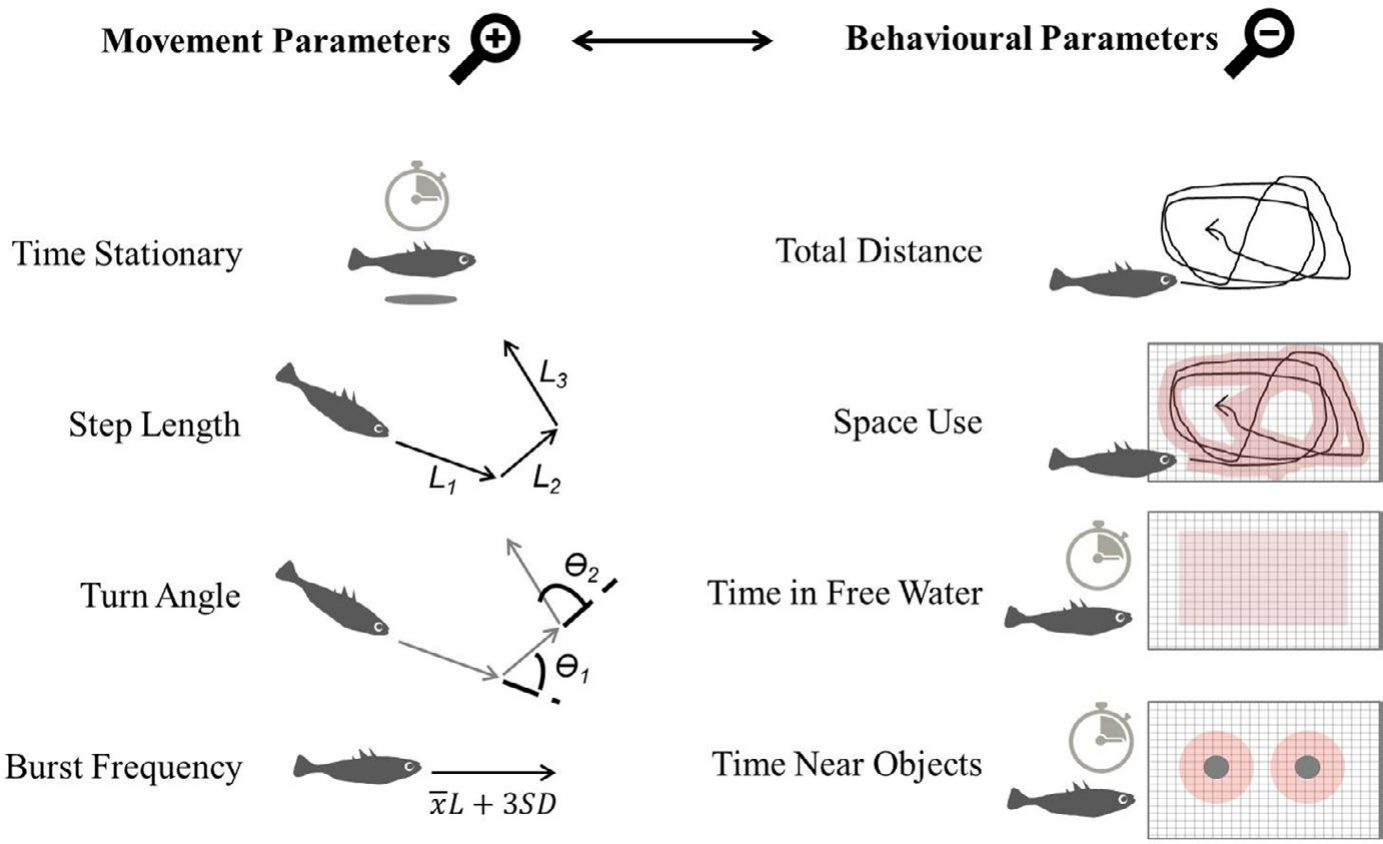
Using high‐resolution tracking data of three‐spined stickleback fish (*Gasterosteus aculeatus*), we examined the repeatability of four movement parameters commonly used in the analysis of discrete time series movement data and four behavioral parameters commonly used in animal personality studies. Movement parameters shown quantify precise characteristics of fish trajectories, while behavioral parameters indicate overall patterns of activity and space use

For each fish and for each trial, we calculated the following movement parameters: (a) Time Stationary (% of trial), (b) Step Length (mean across trial, mm), (c) Turning Angle (mean cosine turn angle, Ө), and (d) Burst Frequency (the relative frequency of periods of movement with a speed above 3 *SD*'s of the mean step length of the fish when moving) (Kane et al., 2004), and the following behavioral parameters: (e) Distance Travelled (total distance travelled during trial) (f) Space Use (proportion of tank two‐dimensional space explored), (g) Time Near Objects (% of time “near” an object during the trial), and (h) Time in Free Water (% time away from tank edges and shelters) (Figure 2). Near (or away) from objects was considered as within 7 cm (larger than fish body length which is on average 5.3 cm in a sample from our study population; Fürtbauer et al., 2015); other distances were considered from 2 to 15 cm, but results were quantitatively similar.

### 2.5 Statistical analyses

All statistical analyses were carried out using R software v3.5.1 (R Core Team, 2018). To assess repeatability in movement and behavioral parameters at the individual level, agreement repeatability was calculated across the six observations per fish (equivalent to the intraclass correlation coefficient (Nakagawa & Schielzeth, 2010; Roche et al., 2016) by fitting a univariate linear mixed model for each parameter using the lme4 package in R (Bates et al., 2015) with only the fish identity (ID) as a random factor (Dingemanse & Dochtermann, 2013; Houslay & Wilson, 2017; Nakagawa & Schielzeth, 2010; Roche et al., 2016). Adjusted repeatability controlling for fixed effects (trial number and environment), was then calculated using the rptR package in R (Nakagawa & Schielzeth, 2013; Stoffel et al., 2017).

To determine whether parameters were correlated between individuals (Hertel et al., 2019), while also testing for the effect of fish identity, and the environment (Houslay & Wilson, 2017; Roche et al., 2016) we fitted a series of mixed models. We fitted each parameter as the response variable (scaled: mean = 0, *SD* = 1), week (1, 2), and environment (two, three, five plants) as fixed effects, and fish ID as a random effect. To compare variation (*V*) attributed to repeatable interindividual differences (ID) and the environment (Env), we modeled ID and ID × Env as random effects (Dingemanse et al., 2010) allowing us to calculate *V*_ID_/*V*_Env_, values close to 0 indicating when *V*_ID_ is negligible compared to the effect of the environment. We also performed an eigendecomposition on the between‐individual covariance matrix to see whether a major axis of among‐individual variation existed (Houslay et al., 2018) indicative of a single latent behavior (Hertel et al., 2019; White et al., 2019).

The mixed model approach described above was fitted using the MCMCglmm package (Hadfield, 2010) and followed the process outlined in Houslay and Wilson (2017) to avoid the anticonservative estimations of best linear unbiased predictors (Hertel et al., 2019; Houslay & Wilson, 2017; Tan & Tan, 2019). We used the MCMCglmm default prior for the fixed effects and an inverse‐gamma prior for the residuals (*V* = 1, *ν* = 0.002). For the random effects an uninformative, parameter‐expanded prior was used with (*V* = 1, *ν* = 0.002, *αμ* = 0, *αV* = 25^2^) (Hertel et al., 2019; Houslay & Wilson, 2017). Posterior distributions were visibly inspected to determine the validity of the algorithms and to ensure convergence, and trace plots (Figure S1) confirmed mixing of chains and absence of autocorrelation between posterior samples (using coda package in R: Plummer et al., 2006). An eigendecomposition on the between‐individual covariance matrix was performed with 95% CIs estimated from 5,000 bootstrapped replicates of the MCMC chain by modifying the bootstrap code provided by Houslay et al. (2018).

## 3 RESULTS

### 3.1 Repeatability of movement and behavioral parameters

Univariate models (Table S1) and mixed effects models (Table S2) revealed consistent interindividual differences in both movement and behavioral parameters, with the exception of Time Near Objects (Table 1). Moreover, the variance explained by fish identity was greater than that explained by changes in the environment, for all parameters except Time in Free Water and Time near Objects (Table 1).

**TABLE 1:** Agreement repeatability estimates (*R*), with 95% confidence intervals (CI) and corresponding *p* value, estimates using the rptR package in R (Stoffel et al., 2017). Variation (V) attributed to repeatable interindividual differences (ID) and the environment (Env), are also provided

Parameter	*R*	95% CI	*p*	*V*_ID_/*V*_Env_
Time stationary	0.43	0.16. 0.62	<.001	0.56
Step length	0.75	0.51, 0.87	<.001	0.74
Turn angle	0.27	0.04. 0.49	.003	0.36
Burst frequency	0.25	0.02, 0.46	.003	0.41
Distance travelled	0.44	0.17, 0.64	<.001	0.91
Space use	0.58	0.30, 0.76	<.001	0.66
Time in free water	0.28	0.04, 0.49	.001	0.01
Time near objects	0.11	0.00, 0.30	.100	0.01

Note

All parameters except for Time Near Objects were repeatable. Comparison of variation (*V*) attributed to repeatable interindividual differences (ID) and the environment (Env) based on output of fitting ID and ID × Env as random effects (Dingemanse et al., 2010) and calculated *V*_ID_/*V*_Env_ are also shown; values close to 0 indicate when *V*_ID_ is negligible. Values imply *V*_ID_ is not negligible compared to *V*_Env_ for all parameters except Time in Free Water and Time near Objects.

### 3.2 Correlations between movement and behavioral parameters

**FIGURE 3:**
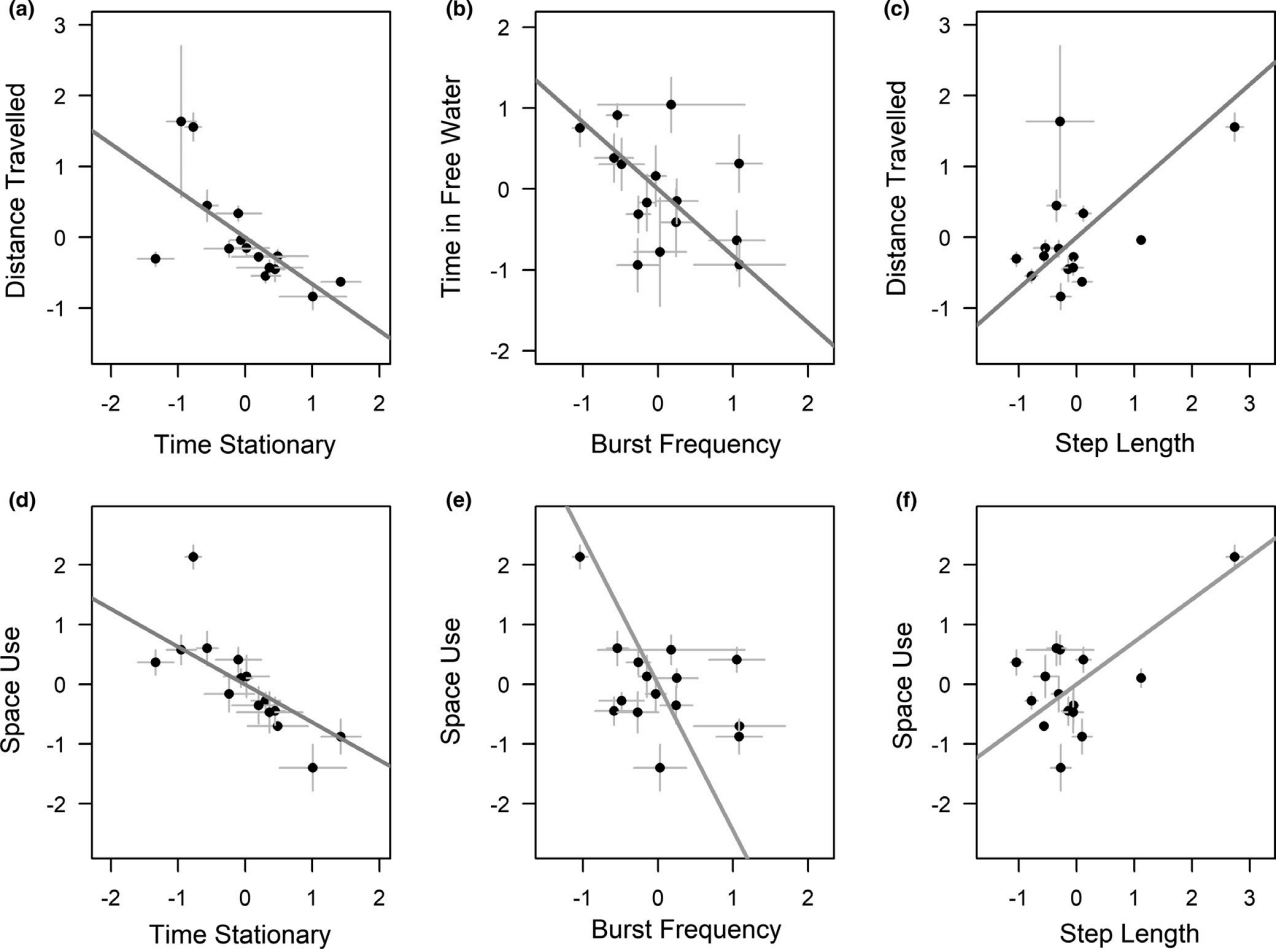
Statistically significant between‐individual correlations among movement parameters and behavioral parameters (Table 2). Parameters are scaled (mean = 0, *SD* = 1) with the dots and error bars representing individual mean averages ± standard error (taken from the posteriors of the random effects from the multivariate MCMCglmm model; Table S2). Regression lines fitted to data are estimated by dividing the covariance of the traits by the variance of the trait on the *x*‐axis (Hertel et al., 2019; Houslay & Wilson, 2017)

We found significant between‐individual correlations among a number of movement parameters and behavioral parameters (Table 2; Figure 3). Time Stationary and Step Length (movement parameters) correlated with Distance Travelled and Space Use (behavioral parameters). Burst Frequency (movement parameter) was correlated with Space Use and Time in Free Water (behavioral parameters). Turning angle (movement parameter) was correlated with Time in Free Water (behavioral parameter).

**TABLE 2:** Adjusted repeatability values (i.e., repeatability values calculated conditioned on the fixed effects) and 95% CIs (in brackets) given in italics across the main diagonal

	Time stationary	Step length	Turn angle	Burst frequency	Distance travelled	Space use	Time in free water
Time stationary	*0.50 (0.23, 0.77)*	−0.08 (−0.68, 0.56)	**−0.65 (−1.00, −0.14)**	**0.67 (0.14, 1.00)**	**−0.66 (−0.97, 0.21)**	**−0.73 (−0.97, −0.36)**	−0.57 (−1.00, 0.02)
Step length		*0.80 (0.66, 0.94)*	0.07 (−0.60, 0.72)	−0.31 (−0.90, 0.34)	**0.50 (0.00, 0.92)**	**0.57 (0.12, 0.94)**	0.32 (−0.32, 0.86)
Turn angle			*0.37 (0.08, 0.65)*	**−0.86 (−1.00, −0.53)**	0.39 (−0.27, 0.96)	0.42 (−0.19, 0.92)	**0.70 (0.16, 1.00)**
Burst frequency				*0.34 (0.07, 0.62)*	−0.53 (−1.00, 0.11)	−0.57 (−0.99, −0.02)	**−0.75 (−1.00, −0.23)**
Distance travelled					*0.53 (0.21, 0.86)*	**0.89 (0.68, 1.00)**	**0.69 (0.18, 1.00)**
Space use						*0.62 (0.39, 0.87)*	**0.64 (0.13, 1.00)**
Time in free water							*0.34 (0.07, 0.64)*

Note

Correlations between parameters along with 95% CI are given above the diagonal. Values are calculated by sampling 4,000 models from the MCMC chain at 1,000‐generation intervals (Hertel et al., 2019). Due to the Bayesian nature of calculating the correlation, values were considered significant if the CIs did not cross 0, and these are shown in bold. Where CIs were close to crossing 0 are underlined.

### 3.3 Major axis of between‐individual variation

Eigendecomposition revealed a major axis of between‐individual variation (Eigenvector 1 = 59.8%, Table S3) representing fish activity/exploration (Eigenvector 1) with behavioral parameters (Distance Travelled, Space Use, Time in Free Water) loading in the same direction as two movement parameters (Turning Angle, Step Length), however the CIs for the movement parameters either straddled or were close to 0 (Table 3). Time Stationary (behavioral parameter) and Burst Frequency (movement parameter) loaded mainly in the opposite direction (Table 3), although the CIs also straddled or were close to 0. Eigenvector 2 was almost entirely loaded by Step Length, and all parameters CIs straddled 0, and hence, no clear conclusions can be made about EV2 (Table 3).

**TABLE 3:** Eigendecomposition on the between‐individual covariance matrix to investigate major axis of among‐individual variation (following Houslay & Wilson, 2017) indicative of a single latent behavior (Hertel et al., 2019; White et al., 2019)

Parameter	EV1	EV2
Time stationary	0.35 (0.01, 0.64)	0.41 (−0.33, 0.78)
Step length	−0.43 (−0.88, −0.01)	0.79 (−0.10, 0.90)
Turn angle	−0.23 (−0.48, 0.05)	−0.36 (−0.75, 0.36)
Burst frequency	0.26 (−0.01, 0.47)	0.20 (−0.44, 0.65)
Distance travelled	−0.45 (−0.67, −0.11)	−0.04 (−0.62, 0.56)
Space use	−0.52 (−0.74, −0.17)	0.01 (−0.62, 0.58)
Time in free water	−0.30 (−0.51, −0.02)	−0.17 (−0.64, 0.43)
Time near objects	0.07 (−0.07, 0.18)	−0.06 (−0.31, 0.25)

## 4 DISCUSSION

We find that stickleback fish show consistent interindividual differences in movement parameters (commonly used in the analysis of discrete movement data) and behavioral parameters (commonly used in animal personality studies), when observed repeatedly across different environments. While our observations of *n* = 15 individuals observed six times is a low sample size (Dingemanse & Dochtermann, 2013), previous studies on this species have shown such behavioral parameters to be repeatable (e.g., Fürtbauer et al., 2015; Jolles et al., 2016, 2018, 2019; King et al., 2013), and movement parameters are repeatable in other fish species (e.g., mosquitofish, *Gambusia holbrooki*: Herbert‐Read et al., 2013). By combining movement and behavioral parameters to quantify the structure of behavioral variation in our study population (White et al., 2019), we show these two approaches—quantifying movement and behavioral parameters—are broadly equivalent.

We demonstrate that movement and behavioral parameters capture similar interindividual variation via correlations among parameters (Figure 3). Furthermore, comparison of the variance accounted for by fish ID and the environment (Table 1) indicates fish ID explains more variation than the environment, for all parameters except for Time in Free Water and Time near Objects which are determined by the environment directly, and not by fish behavior/movement. Eigendecomposition on the between‐individual covariance also suggests a single axis of between‐individual variation representing activity/exploration (Table 3). Specifically, we find that fish that spent less time stationary, tookmore direct paths, and less commonly burst travelled (movement parameters), were also observed to travel farther, explore more of the tank, and spend more time in open water (behavioral parameters). However, the CIs for the loading of movement parameters are larger than those of behavioral parameters (Table 3) suggesting movement parameters may be less reliable measures of this activity/exploration axis. Studies investigating correlations among movement and behavioral parameters in other species and contexts are therefore needed. Nevertheless, we expect the consistent interindividual differences in movement parameters and their correlation with behavioral parameters to represent a general phenomenon (Spiegel et al., 2017) with implications for personality and movement ecology research (Nathan et al., 2008; Schick et al., 2008; Spiegel et al., 2017) as discussed below.

Laboratory studies of animal personality make observations of individuals over many minutes or hours (e.g., studies of fish or insects) and often disregard data from an arbitrarily defined period at the start of observations to allow subjects to acclimatize to the test arena or circumstances. Our findings indicate that researchers may be able to use movement parameters (e.g., time spent stationary and burst frequency) to not only quantitatively determine acclimatization periods, but also assay personality types using minimal trajectory data. In the case of determining acclimatization periods, moving average calculations and change‐point tests (Picard, 1985) will allow researchers to define periods during which movement parameters are consistent. In practice, this will likely differentiate time periods at the start of trials from the rest of the observation. Data over seconds during identified stable periods may also be sufficient to assay an individual's personality type (sensu David et al., 2012; MacKay et al., 2013). Furthermore, if this process could be automated by a tracking system (e.g., Alarcon‐Nieto et al., 2018; Dell et al., 2014; Matthews et al., 2017; Strömbom & King, 2018), it would open the possibility for generating large and robust datasets affording studies linking individual differences in behavior to evolutionary processes (e.g., Alarcon‐Nieto et al., 2018; Gernat et al., 2018; King et al., 2018; Rudolf et al., 2019; Sabol et al., 2018; Valletta et al., 2017), or animal welfare and management (e.g., Fehlmann et al., 2017; Henry et al., 2018; Matthews et al., 2017). Note, however, the discussion above would only be applicable to relatively fast‐moving species and not be applicable to, for example, studies of personality in gastropods (e.g., Ahlgren et al., 2015).

With the increasing availability of telemetry data (Kranstauber et al., 2011; Krause et al., 2013), movement parameters may also provide a robust way to analyze personality traits for species that are difficult or impossible to study using standardized behavioral assays (Carter et al., 2013), allowing further integration of movement ecology with other fields of behavioral ecology (Hertel et al., 2020). For example, an advantage of such in situ movement data is that researchers can examine changes in movement parameters at an individual to quantify flexibility in personality (e.g., Betini & Norris, 2012; Briffa et al., 2008; Carere et al., 2005; Carter et al., 2013; Dingemanse et al., 2010; Frost et al., 2007; Kralj‐Fiser & Schneider, 2012; Quinn & Cresswell, 2005). Statistically, this involves testing individuals' plasticity or “reaction norms” to different environments or contexts (Araya‐Ajoy, Mathot, & Dingemanse, 2015; Cornwell, McCarthy, Snyder, & Biro, 2019; Dingemanse & Dochtermann, 2013). While this requires large sample sizes (numbers of individuals), tracking the movements of many individuals simultaneously is now possible in the wild (King et al., 2018). Future work can therefore adopt a behavioral type‐based approach to understand the consequences of fixed or flexible behaviors at the individual level for population dynamics and the emergence of complex ecological patterns (e.g., Getz et al., 2018; Spiegel et al., 2017). There is increasing evidence for personality‐dependent space use (e.g., Schirmer et al., 2019), and experiments with *Tribolium confusum* beetles, for example, found that conventional correlated random walk models, which do not incorporate interindividual differences in movement, were unable to account for the authors' data in a series of landscape experiments (Morales & Ellner, 2002).

Our case study supports a proposal for movement studies to acknowledge and study consistent individual differences, and, conversely, animal personality studies to adopt a more quantitative representation of movement patterns (e.g., Getz et al., 2018; Spiegel et al., 2017). Indeed, for researchers interested in “higher order” group‐ and population‐level behaviors, it is necessary to incorporate such individual‐level variation into their studies (King et al., 2018). However, where individual‐level data are collected in isolation (i.e., solitary individuals) we urge caution using these data to build data‐ or theory‐driven movement models, since variation in the social environment can profoundly alter the expression of movement and behavior (e.g., Fürtbauer & Fry, 2018; Herbert‐Read et al., 2013; King et al., 2015; Zhang et al., 2020), and this poses a new challenge for researchers in both areas. In short, the two research areas should continue to collaborate to advance their respective and combined fields of research.

## CONFLICT OF INTEREST

None declared.

## AUTHOR CONTRIBUTION

**Joseph D. Bailey:** Data curation (equal); Formal analysis (lead); Visualization (lead); Writing‐review & editing (supporting). **Andrew J. King:** Conceptualization (supporting); Data curation (supporting); Formal analysis (supporting); Funding acquisition (equal); Investigation (supporting); Methodology (supporting); Project administration (supporting); Resources (equal); Supervision (lead); Writing‐original draft (lead). **Edward A. Codling:** Formal analysis (supporting); Project administration (supporting); Writing‐review & editing (supporting). **Ashley M. Short:** Data curation (equal); Investigation (equal); Methodology (equal). **Gemma I. Johns:** Data curation (equal); Investigation (equal); Methodology (equal). **Ines Fürtbauer:** Conceptualization (lead); Data curation (supporting); Funding acquisition (equal); Investigation (equal); Project administration (equal); Resources (equal); Supervision (supporting); Writing‐original draft (supporting); Writing‐review & editing (supporting).

## ETHICAL APPROVAL

This work was approved by Swansea University institutional Animal Welfare and Ethical Review Body (AWERB) REF‐IP‐1213‐3.

## Supporting information

Supplementary Material

Data S1

## Data Availability

All data needed to perform analyses uploaded as supplementary material.
