# Supplementary material for: “Micropersonality” traits and their implications for behavioral and movement ecology research
Source: Ecol Evol. 2021 Feb 22;11(7):3264–73. doi: 10.1002/ece3.7275 (PMC8019044; doi:10.1002/ece3.7275)
Supplement: Supplementary file 1 — Supplementary Material [file ECE3-11-3264-s002.docx]

**SUPPLEMENTARY MATERIAL**

**Table S1** – Estimated fixed-effect sizes in linear mixed models using the lme4 package in R (Bates et al, 2014) with only the fish ID as a random factor

|  | **Intercept** | **Std. Error** | **t value** | **Pr(>\|t\|)** |
| --- | --- | --- | --- | --- |
| Time Stationary | 0.3781 | 0.0141 | 26.91 | <0.001 |
| Step Length | 14.92 | 0.4288 | 34.8 | <0.001 |
| Turn Angle | 0.7329 | 0.01 | 73.45 | <0.001 |
| Burst Frequency | 0.008 | 0.0006 | 13.84 | <0.001 |
| Distance Travelled | 31410 | 1835 | 17.11 | <0.001 |
| Space Use | 0.3186 | 0.0112 | 28.5 | <0.001 |
| Time in Free Water | 0.545 | 0.0163 | 33.46 | <0.001 |
| Time Near Objects | 0.1613 | 0.0144 | 11.23 | <0.001 |

**Table S2 –** Estimated fixed-effect sizes in the multivariate mixed model. Each parameter was considered as the response variable, the trial number and environment (categorical: two, three, five plants) as fixed effects, and fish ID as a random effect. All parameters were scaled (mean=0, s.d.=1)**.**

|  | **Estimate** | **lower interval** | **upper interval** | **pMCMC** |
| --- | --- | --- | --- | --- |
| **Time Stationary** |  |  |  |  |
| (Intercept) | 0.0230 | -0.4635 | 0.4978 | 0.9142 |
| Environment | -0.1819 | -0.3828 | 0.0302 | 0.0783 |
| Week | -0.4401 | -0.7947 | -0.1391 | 0.0111 |
| **Step Length** |  |  |  |  |
| (Intercept) | -0.0180 | -0.6152 | 0.6332 | 0.9506 |
| Environment | -0.0030 | -0.1415 | 0.1400 | 0.9637 |
| Week | -0.1357 | -0.3701 | 0.0709 | 0.2338 |
| **Turn Angle** |  |  |  |  |
| (Intercept) | -0.0353 | -0.4508 | 0.3927 | 0.8536 |
| Environment | 0.0137 | -0.2437 | 0.2255 | 0.8843 |
| Week | 0.4322 | 0.0576 | 0.8055 | 0.0248 |
| **Burst Frequency** |  |  |  |  |
| (Intercept) | 0.0257 | -0.4307 | 0.3815 | 0.8673 |
| Environment | 0.1600 | -0.0882 | 0.3771 | 0.1982 |
| Week | -0.3278 | -0.7170 | 0.0699 | 0.1030 |
| **Distance Travelled** |  |  |  |  |
| (Intercept) | -0.0098 | -0.5198 | 0.4704 | 0.9540 |
| Environment | 0.0867 | -0.1121 | 0.2972 | 0.4134 |
| Week | -0.3833 | -0.7267 | -0.0659 | 0.0189 |
| **Space Use** |  |  |  |  |
| (Intercept) | -0.0121 | -0.5594 | 0.4965 | 0.9449 |
| Environment | -0.1036 | -0.2797 | 0.0884 | 0.2713 |
| Week | 0.0264 | -0.2738 | 0.3172 | 0.8699 |
| **Time in Free Water** |  |  |  |  |
| (Intercept) | -0.0257 | -0.4362 | 0.3682 | 0.8751 |
| Environment | -0.1442 | -0.3847 | 0.1052 | 0.2400 |
| Week | 0.2316 | -0.1754 | 0.6318 | 0.2256 |

**Table S3** – Results of Eigen analysis. Eigenvector 1 (EV1) was seen to account for almost 60% of the observed among individual variation, possibly indicating the presence of a major axis of variation, which can be interpreted as a latent behavioural axis (Houslay et al, 2017)

|  | % | Eigenvalue |
| --- | --- | --- |
| EV1 | 59.8% | 3.28 |
| EV2 | 22.4% | 1.23 |
| EV3 | 8.8% | 0.48 |
| EV4 | 3.5% | 0.19 |
| EV5 | 1.8% | 0.10 |
| EV6 | 1.5% | 0.08 |
| EV7 | 1.2% | 0.07 |
| EV8 | 1.0% | 0.05 |

**Figure S1.** Diagnostic trace and density plots of the posterior distributions found when fitting the mixed effect model for the between-individual correlation of parameters


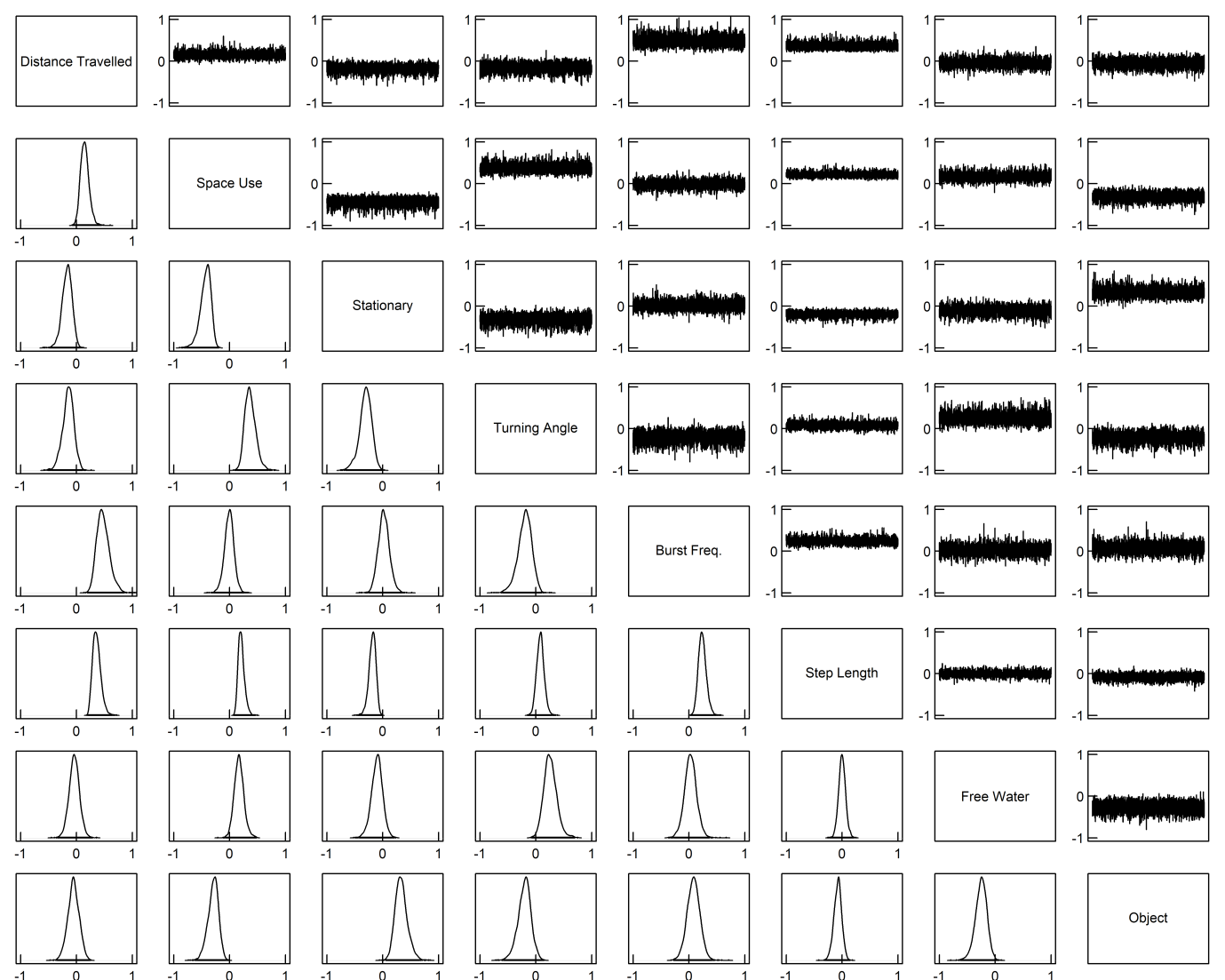


**The movement threshold and sub-sampling rates**

The movement threshold and sub-sampling rates we used (Methods) are in essence arbitrary values but were chosen to retain as much information about the movement path, whilst minimising any causal effects such smoothing can have on characteristics of movement trajectories (Bovet & Benhamou, 1988; Codling & Hill, 2005; Gurarie & Ovaskainen 2011; Benhamou 2014; Bailey et al, 2020). To ensure that different combinations of thresholds and subsampling values did not affect our findings here we present scatterplots (Figures S2-S10) for different movement parameters for varying speed threshold (2mm/s, 5mm/s, 10mm/s) and sampling rates (5Hz, 2.5Hz, 1Hz). Plots demonstrate there is no qualitative difference in the relationship between the parameters regarding the choice of sampling rate and threshold.


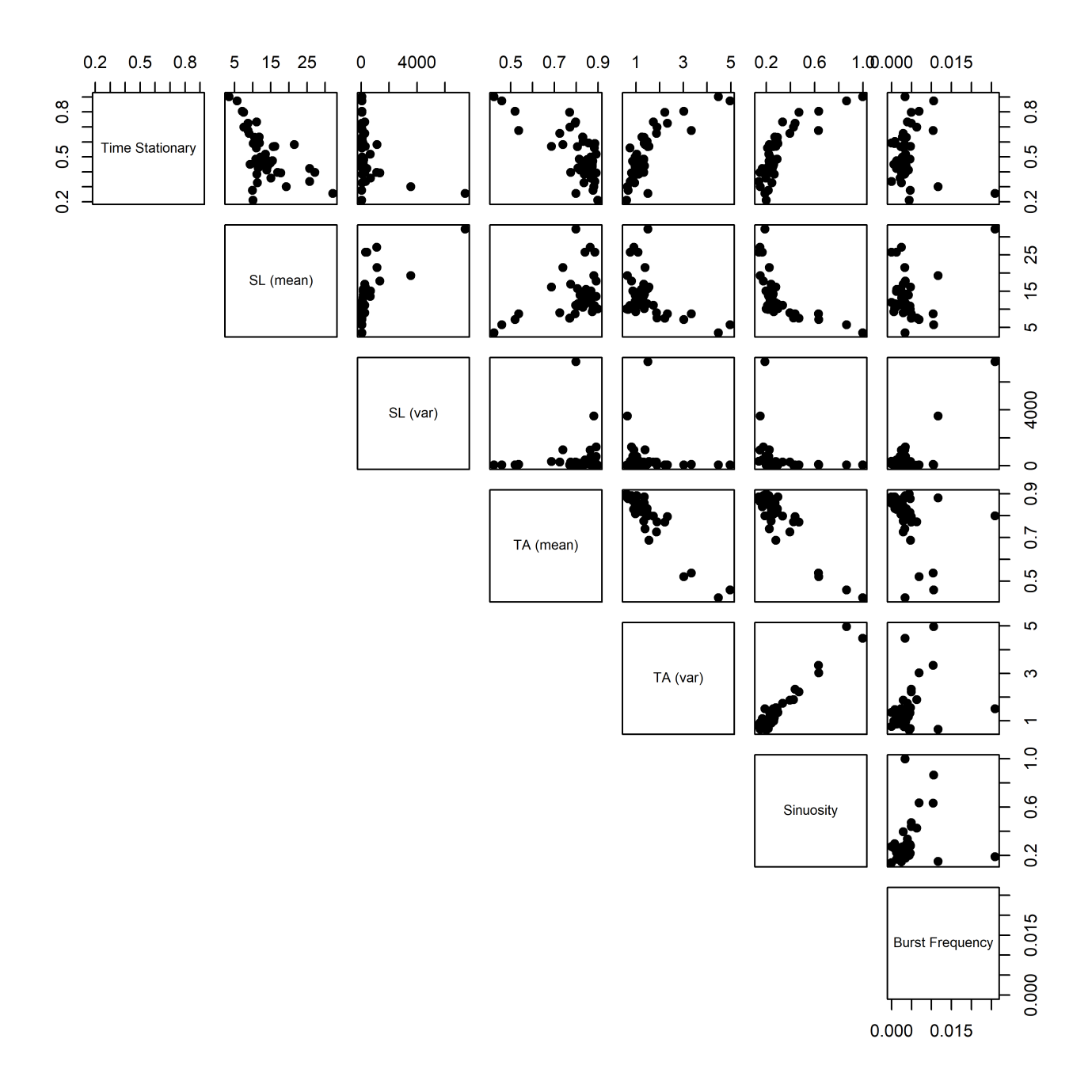


**Figure S2.** Threshold 2mm/s, sampling rate 5Hz. Data are presented for Time Stationary, Step Length (mean), Step Length (median), Turn Angle (Mean), Turn Angle (Variance), Sinuosity, and Burst Frequency.


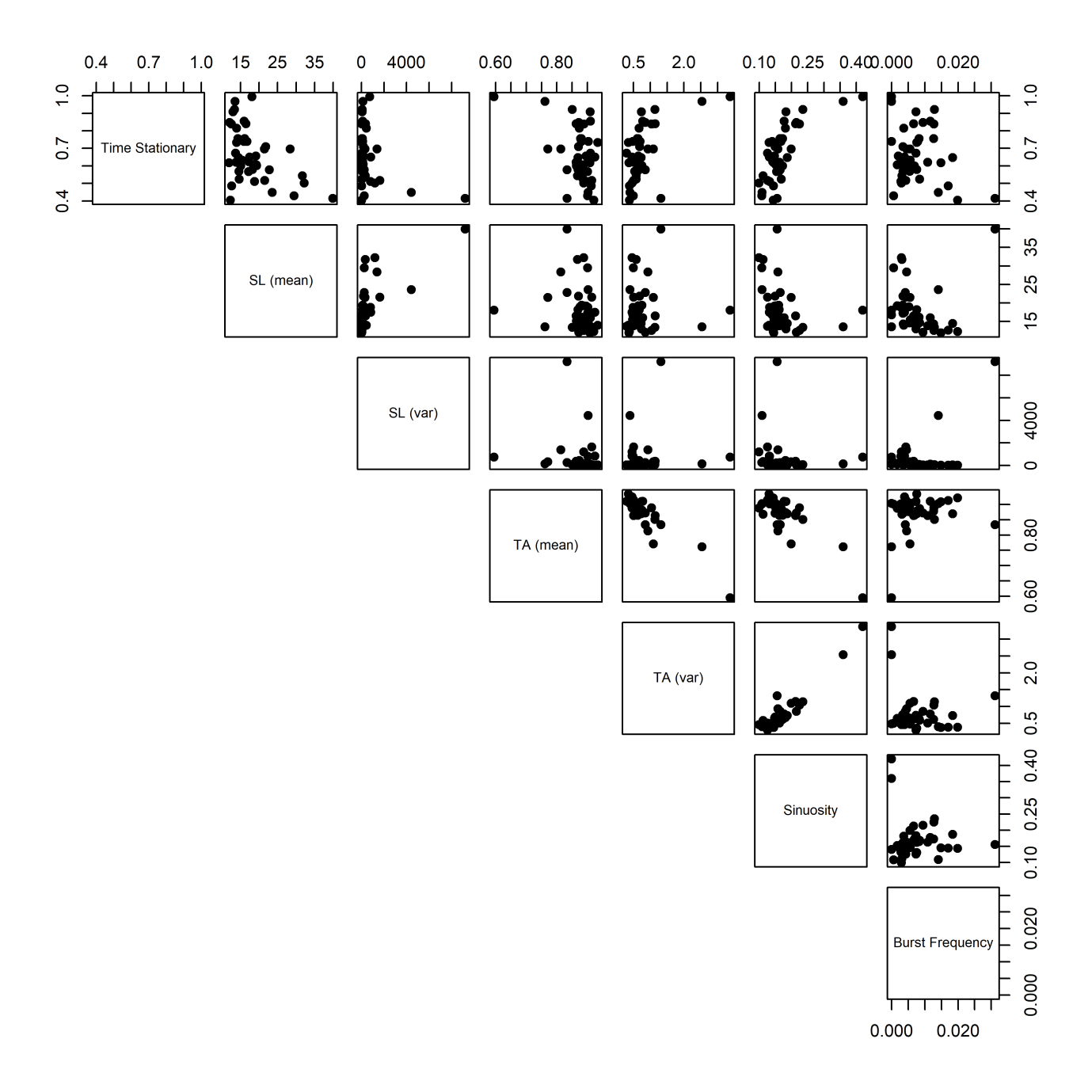


**Figure S3.** Threshold 5mm/s, sampling rate 5Hz. Data are presented for Time Stationary, Step Length (mean), Step Length (median), Turn Angle (Mean), Turn Angle (Variance), Sinuosity, and Burst Frequency.


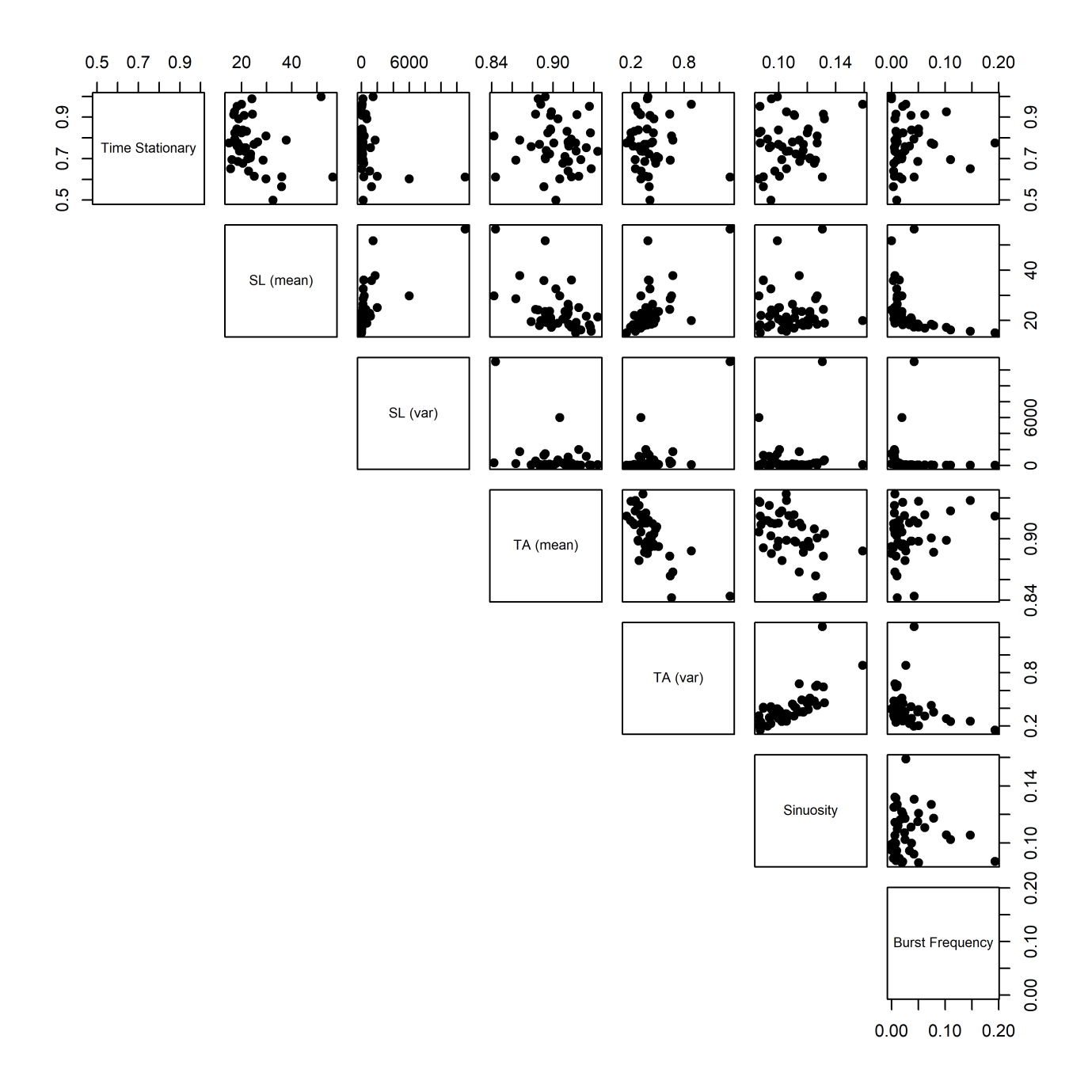


**Figure S4.** Threshold 10mm/s, sampling rate 5Hz. Data are presented for Time Stationary, Step Length (mean), Step Length (median), Turn Angle (Mean), Turn Angle (Variance), Sinuosity, and Burst Frequency.


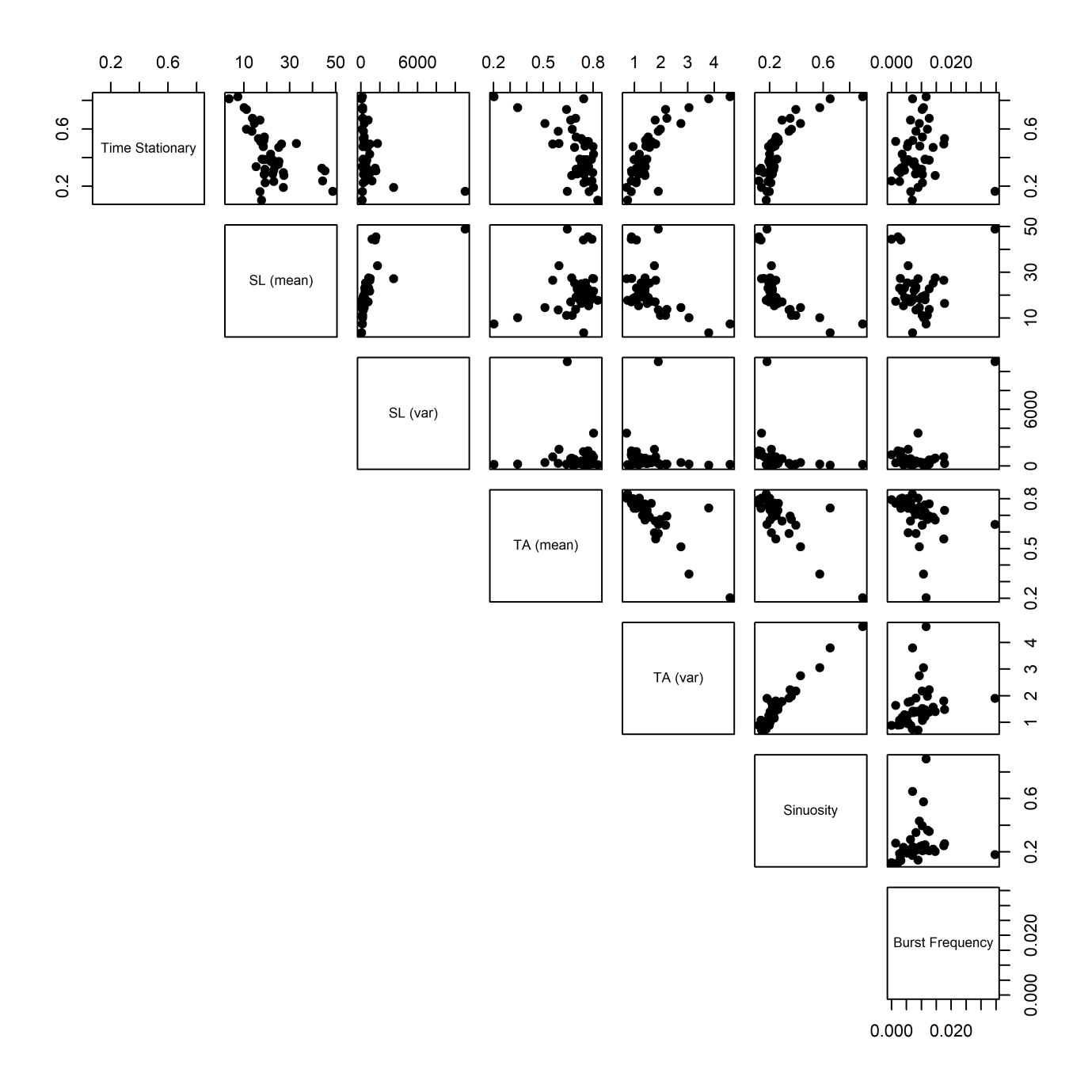


**Figure S5**. Threshold 2mm/s, sampling rate 2.5Hz. Data are presented for Time Stationary, Step Length (mean), Step Length (median), Turn Angle (Mean), Turn Angle (Variance), Sinuosity, and Burst Frequency.


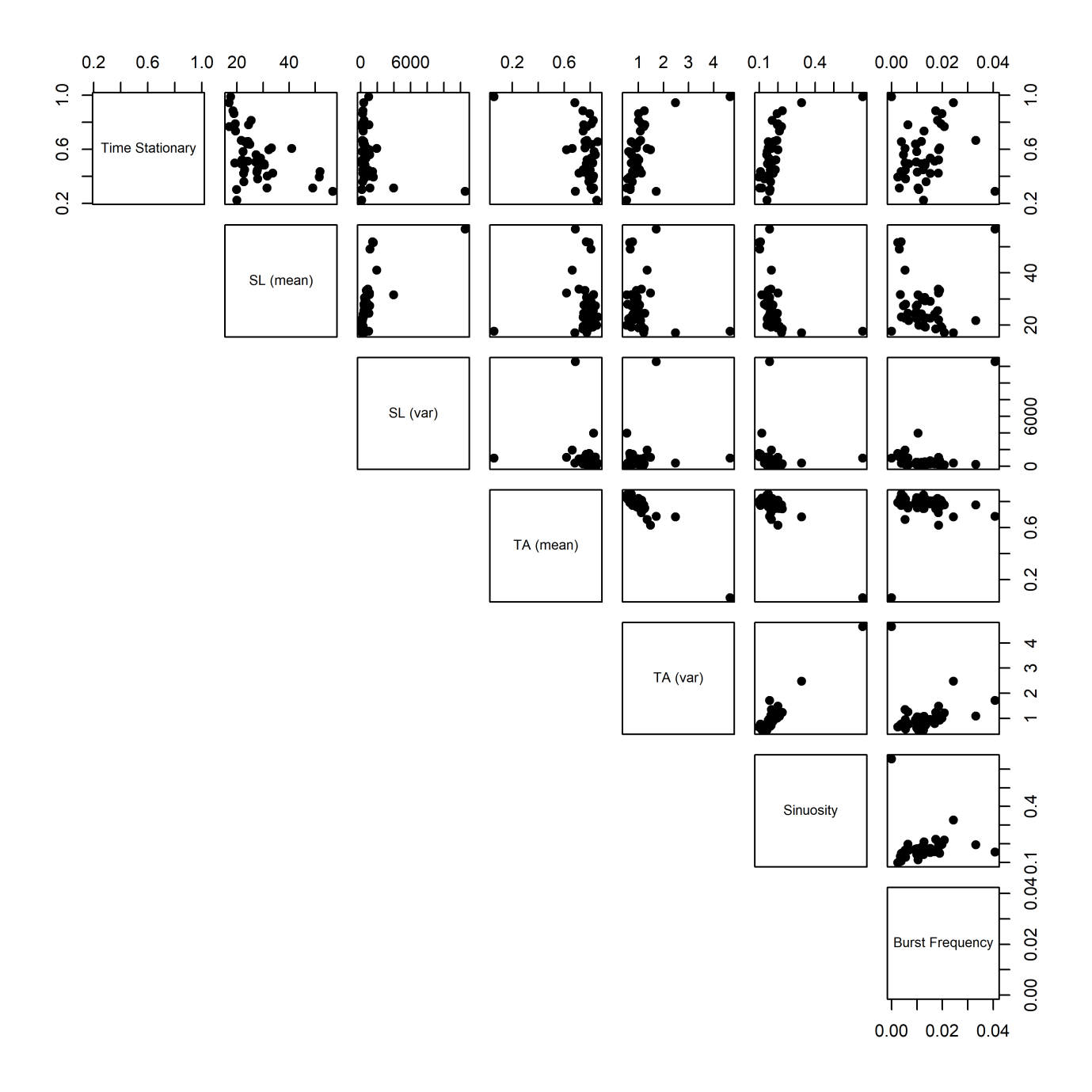


**Figure S6.** Threshold 5mm/s, sampling rate 2.5Hz. Data are presented for Time Stationary, Step Length (mean), Step Length (median), Turn Angle (Mean), Turn Angle (Variance), Sinuosity, and Burst Frequency.


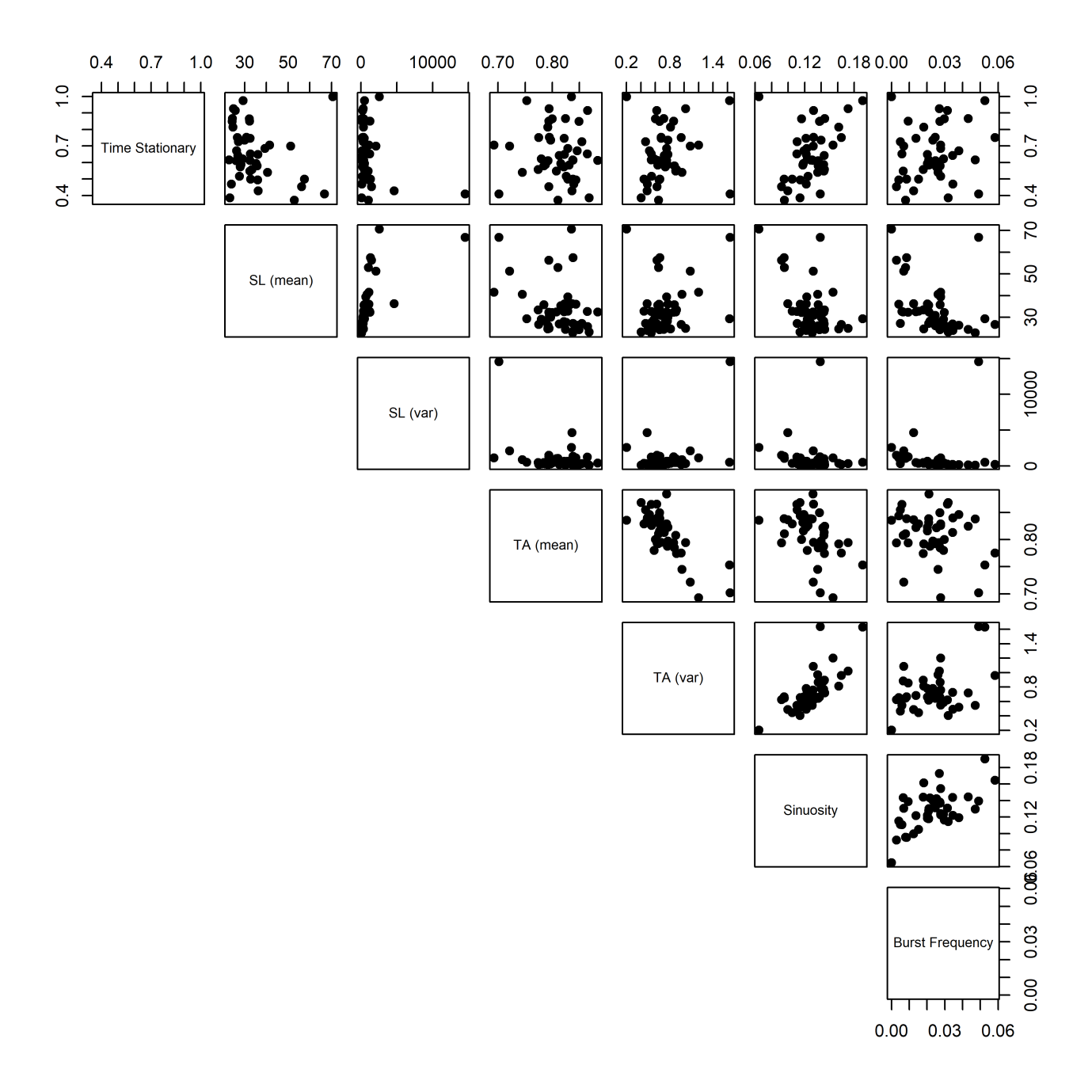


**Figure S7.** Threshold 10mm/s, sampling rate 2.5Hz. Data are presented for Time Stationary, Step Length (mean), Step Length (median), Turn Angle (Mean), Turn Angle (Variance), Sinuosity, and Burst Frequency.


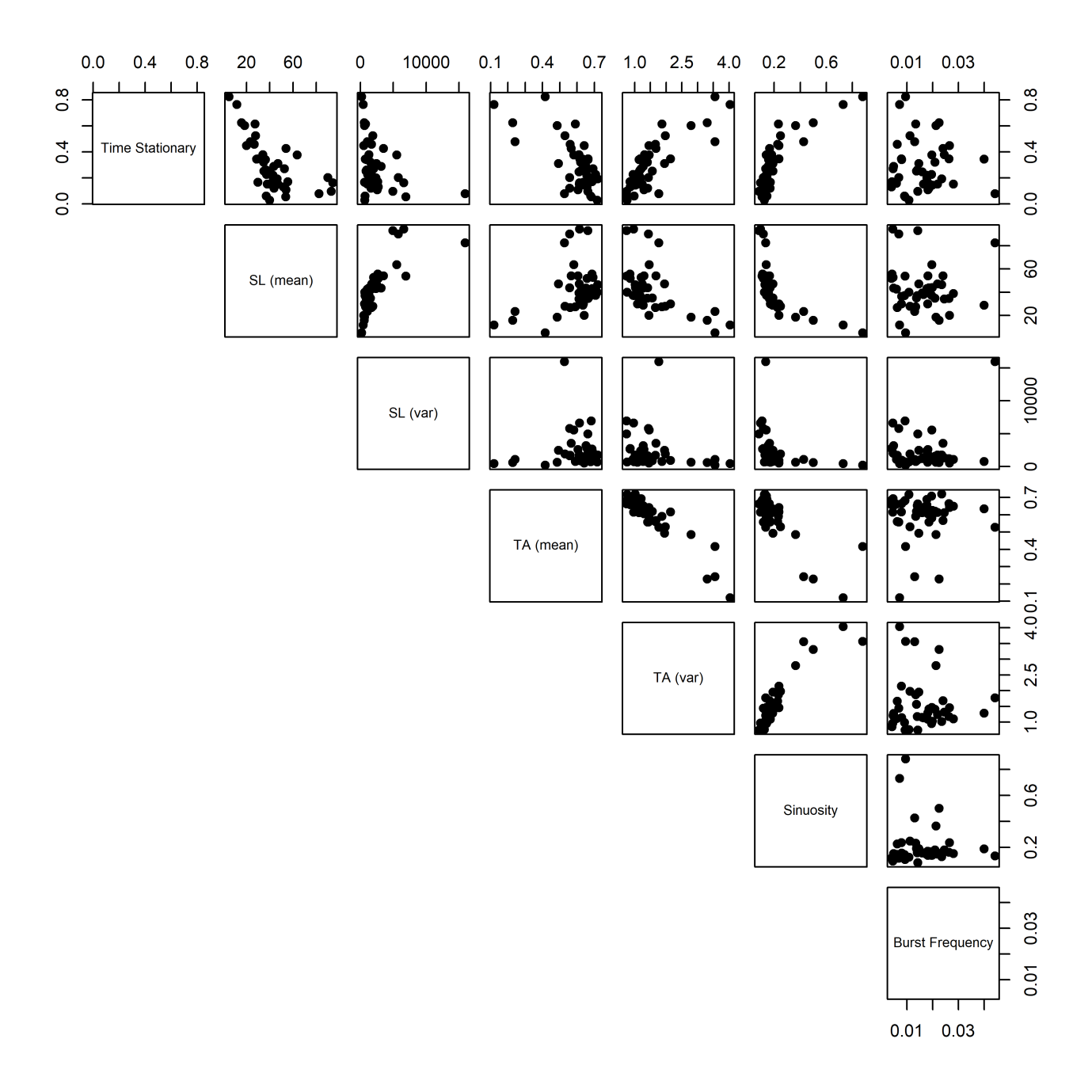


**Figure S8.** Threshold 2mm/s, sampling rate 1Hz. Data are presented for Time Stationary, Step Length (mean), Step Length (median), Turn Angle (Mean), Turn Angle (Variance), Sinuosity, and Burst Frequency.


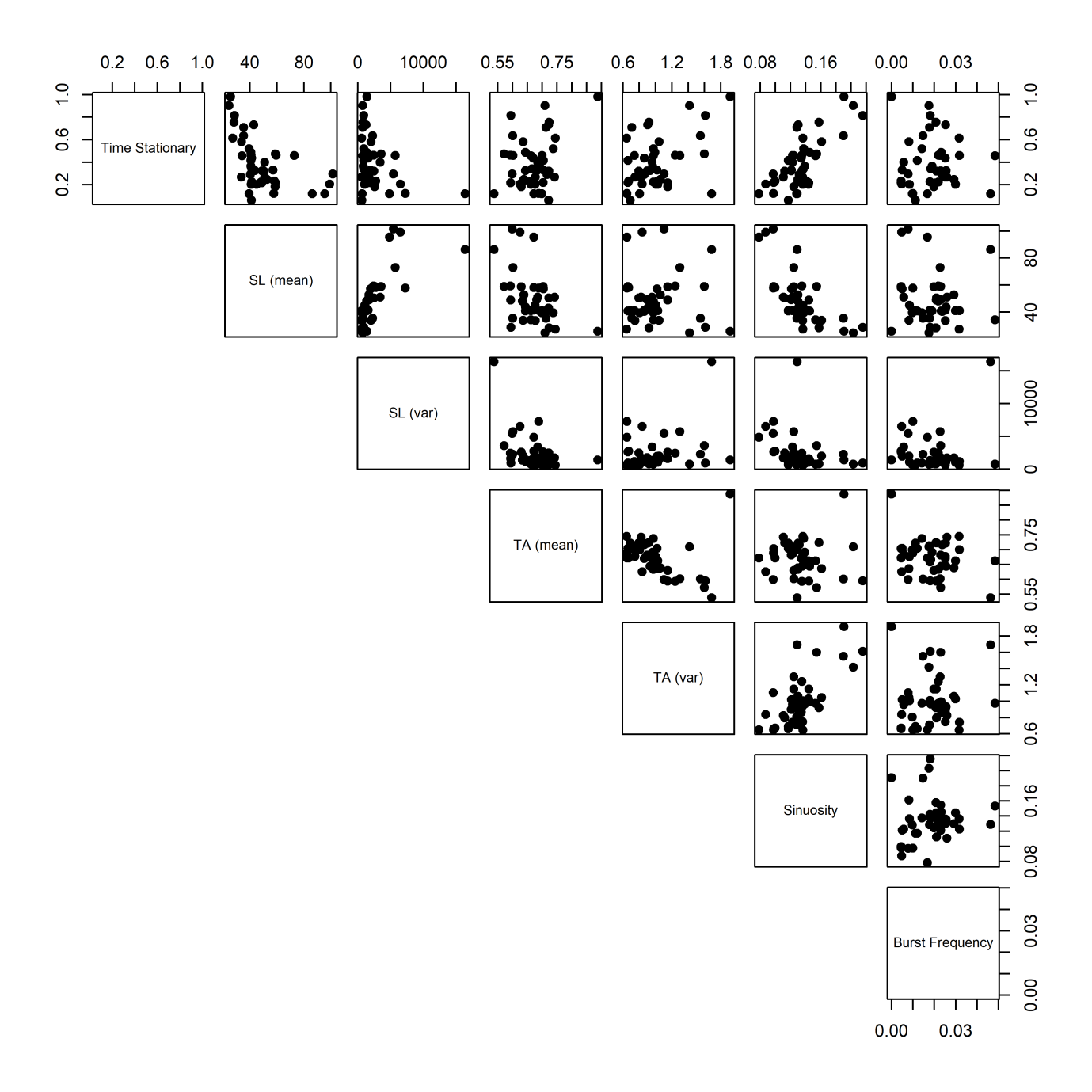


**Figure S9.** Threshold 5mm/s, sampling rate 1Hz. Data are presented for Time Stationary, Step Length (mean), Step Length (median), Turn Angle (Mean), Turn Angle (Variance), Sinuosity, and Burst Frequency.


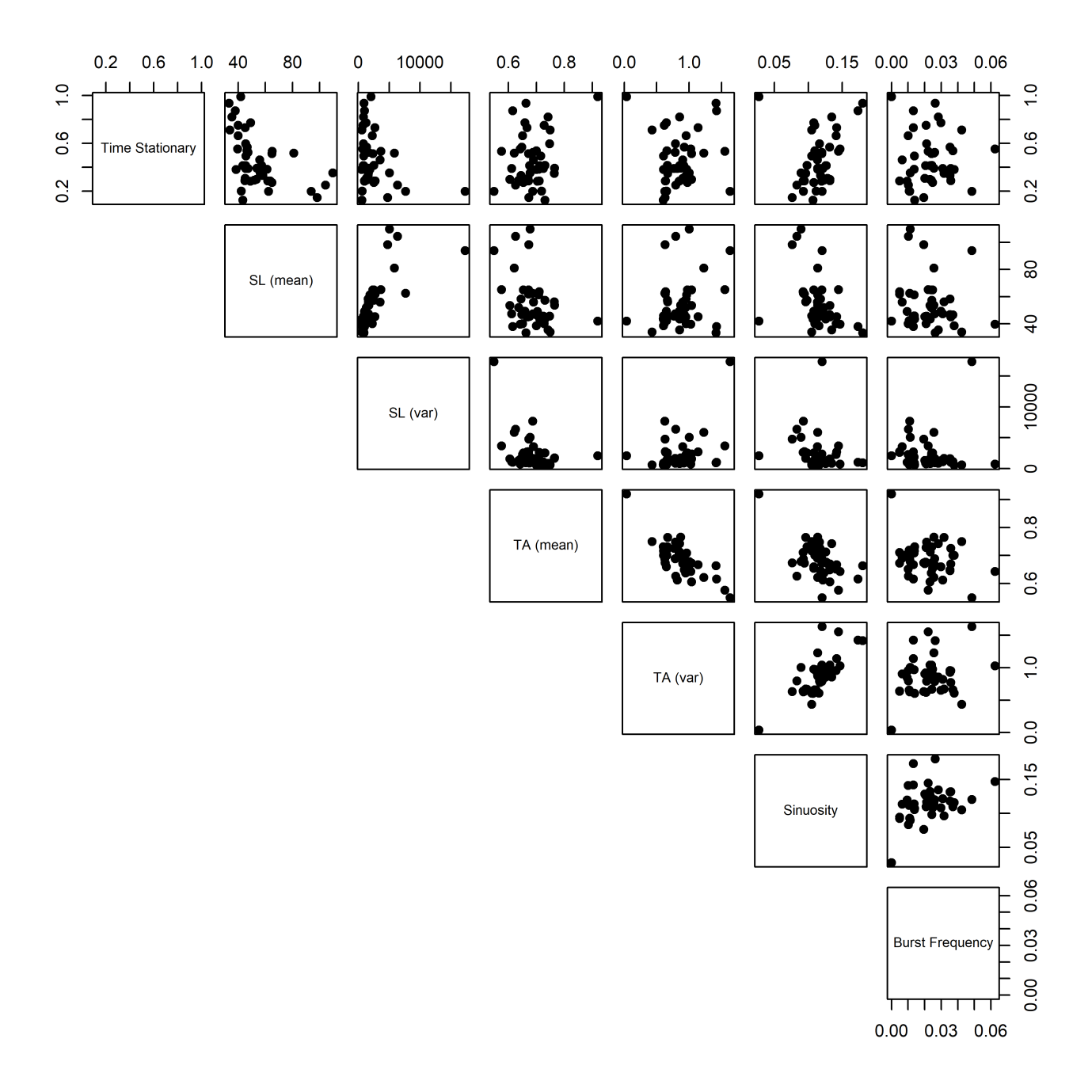


**Figure S10.** Threshold 10mm/s, sampling rate 1Hz. Data are presented for Time Stationary, Step Length (mean), Step Length (median), Turn Angle (Mean), Turn Angle (Variance), Sinuosity, and Burst Frequency.
